# Some Synonymous and Nonsynonymous *gyrA* Mutations in Mycobacterium tuberculosis Lead to Systematic False-Positive Fluoroquinolone Resistance Results with the Hain GenoType MTBDR*sl* Assays

**DOI:** 10.1128/AAC.02169-16

**Published:** 2017-03-24

**Authors:** Adebisi Ajileye, Nataly Alvarez, Matthias Merker, Timothy M. Walker, Suriya Akter, Kerstin Brown, Danesh Moradigaravand, Thomas Schön, Sönke Andres, Viola Schleusener, Shaheed V. Omar, Francesc Coll, Hairong Huang, Roland Diel, Nazir Ismail, Julian Parkhill, Bouke C. de Jong, Tim E. A. Peto, Derrick W. Crook, Stefan Niemann, Jaime Robledo, E. Grace Smith, Sharon J. Peacock, Claudio U. Köser

**Affiliations:** a Public Health England West Midlands Public Health Laboratory, Heartlands Hospital, Birmingham, United Kingdom; b Bacteriology and Mycobacteria Unit, Corporación Para Investigaciones Biológicas, Medellín, Colombia; c Universidad Pontificia Bolivariana, Medellín, Colombia; d Division of Molecular and Experimental Mycobacteriology, Research Center Borstel, Borstel, Germany; e German Center for Infection Research (DZIF), Partnersite Hamburg-Lübeck-Borstel, Borstel, Germany; f Nuffield Department of Medicine, University of Oxford, John Radcliffe Hospital, Oxford, United Kingdom; g Mycobacteriology Unit, Department of Microbiology, Institute of Tropical Medicine, Antwerp, Belgium; h Wellcome Trust Sanger Institute, Hinxton, United Kingdom; i Department of Clinical and Experimental Medicine, Division of Medical Microbiology, Linköping University, Linköping, Sweden; j Department of Clinical Microbiology and Infectious Diseases, Kalmar County Hospital, Kalmar, Sweden; k Division of Mycobacteriology, National Tuberculosis Reference Laboratory, Research Center Borstel, Borstel, Germany; l Centre for Tuberculosis, National Institute for Communicable Diseases, Johannesburg, South Africa; m London School of Hygiene & Tropical Medicine, London, United Kingdom; n National Clinical Laboratory on Tuberculosis, Beijing Key Laboratory on Drug-Resistant Tuberculosis Research, Beijing Chest Hospital, Capital Medical University, Beijing Tuberculosis and Thoracic Tumor Institute, Beijing, China; o Institute of Epidemiology, University Hospital Schleswig-Holstein, Campus Kiel, Kiel, Germany; p Public Health England, Microbiology Services, London, United Kingdom; q Department of Medicine, University of Cambridge, Cambridge, United Kingdom

**Keywords:** Mycobacterium tuberculosis, Hain GenoType MTBDR*sl*, fluoroquinolones

## Abstract

In this study, using the Hain GenoType MTBDR*sl* assays (versions 1 and 2), we found that some nonsynonymous and synonymous mutations in *gyrA* in Mycobacterium tuberculosis result in systematic false-resistance results to fluoroquinolones by preventing the binding of wild-type probes. Moreover, such mutations can prevent the binding of mutant probes designed for the identification of specific resistance mutations. Although these mutations are likely rare globally, they occur in approximately 7% of multidrug-resistant tuberculosis strains in some settings.

## TEXT

As part of its recommendation for a shorter treatment regimen for multidrug-resistant tuberculosis (MDR TB), the World Health Organization (WHO) recently endorsed version 2 of the Hain GenoType MTBDR*sl* as the first genotypic drug susceptibility testing (DST) assay for detecting resistance to fluoroquinolones and to the second-line injectable drugs kanamycin, amikacin, and capreomycin (1–5). Specifically, the WHO has endorsed its use instead of phenotypic methods as an initial direct test for ruling in resistance in patients with either MDR TB or confirmed resistance to rifampin. The precise correlation between genotype and phenotype for some mutations, however, remains unclear, which complicates the interpretation of this assay (5). The WHO is currently reviewing the available evidence to address this point.

**FIG 1:**
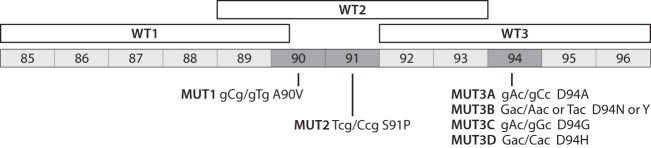
Line probe assays consist of oligonucleotide probes that are immobilized on a nitrocellulose strip. This diagram depicts the region of *gyrA* targeted by the MTBDR*sl* assay (numbers refer to codons). The binding of a mutant probe (MUT1-3D) that targets the three codons highlighted in dark gray (90, 91, and 94; the corresponding nucleotide and amino acid changes are shown under the respective codons) and/or lack of binding of a wild-type probe (WT1-3) is interpreted as genotypic fluoroquinolone resistance, provided that all control bands of the assay, including the one for *gyrA*, are positive. The diagram was based on the package insert of version 1 of the assay (40). The exact design of the wild-type probes is regarded as a trade secret by Hain Lifescience, so it is unclear whether the WT3 band covers all three nucleotides of codon 92. The mutant probes cannot be depicted, as they also constitute a trade secret. Versions 1 and 2 of the assay are identical with regard to the *gyrA* region; thus, results from version 1, which was used for most experiments in this study, should also be valid for version 2 (4).

The only documented instance of systematic false-positive fluoroquinolone resistance results with the MTBDR*sl* was caused by the *gyrA* Acc/Gcc T80A gCg/gGg A90G double mutations relative to the Mycobacterium tuberculosis H37Rv laboratory strain, given that the A90G mutation prevents the binding of the WT2 band of this assay (Fig. 1) (6–9). Several independent studies, which used a variety of techniques, demonstrated that these double mutations do not confer resistance to any of the four fluoroquinolones currently used for the treatment of TB (i.e., ofloxacin, levofloxacin, moxifloxacin, and gatifloxacin) and may even result in hypersusceptibility (6, 7, 9–15). Unfortunately, most of the strains with double mutants were not typed, which left two key questions largely unanswered. First, it remains unclear whether these strains are monophyletic or polyphyletic. Second, there is only limited evidence on how widespread the group(s) of strains with these mutations is.

There are several pieces of circumstantial evidence regarding these mutations. Only 10 primary research studies from our internal database of 265 in which *gyrA* was studied reported these double mutations, although it should be noted that not all of these studies covered codon 80 (6–15). This suggested that these mutations are not widespread globally. Based on studies that found the T80A mutation to be a marker for the M. tuberculosis Uganda genotype (formerly known as Mycobacterium africanum subtype II but now known to be a sublineage within Euro-American M. tuberculosis lineage 4), we speculated that the *gyrA* double mutant strains might constitute a subgroup of the Uganda genotype (16, 17). This hypothesis appeared to be consistent with the results of two studies from the Republic of the Congo and the Democratic Republic of the Congo, which reported the highest frequency of these double mutants (in 60% [9/15] versus 7.2% [15/209] of MDR TB cases from Brazzaville and Pointe-Noire versus Kinshasa, respectively) (7, 8). This was further supported by mycobacterial interspersed repetitive-unit–variable-number tandem-repeat (MIRU-VNTR) results (7, 15).

**FIG 2:**
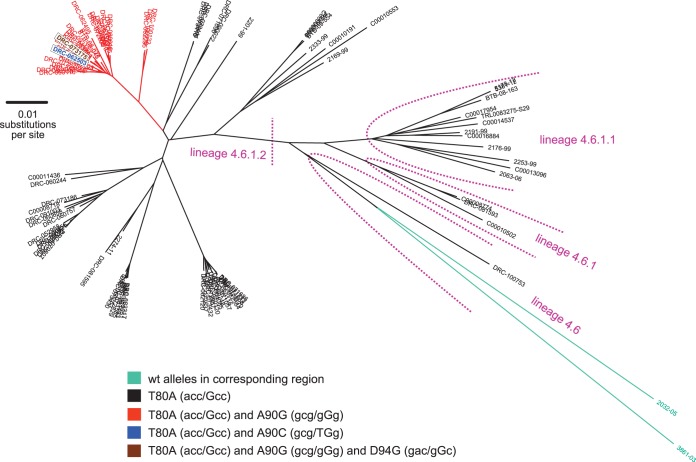
Maximum likelihood phylogeny based on 3,710 single nucleotide variants differentiating all 95 Uganda and Uganda-like M. tuberculosis strains. The numerical code shown corresponds to the lineage classification by Coll et al. (41). Phylogenetic variants in the *gyrA* fluoroquinolone resistance-determining region are color coded. The 28 T80A+A90G strains (or variants thereof) formed a monophyletic group and were consistently susceptible to ofloxacin and other fluoroquinolones when tested (see Table S1 in the supplemental material). This group included the novel T80A+A90C double mutant and, importantly, the T80A+A90G+D94G triple mutant, which comprised the high-confidence D94G resistance mutation that was genetically linked to the double mutations (as opposed to occurring in the same population as a mixed infection) (12). This was in line with a recent report by Pantel et al., who suggested that classical resistance mutations may not cause resistance in a T80A+A90G background, whereas a study by Brossier et al. found that this combination of mutations did correlate with ofloxacin resistance (6, 15). It is therefore possible that these triple mutants have MICs close to the epidemiological cutoff value for ofloxacin, although more data are required to confirm this hypothesis (42, 43).

To clarify the exact relationship of these double mutants with regard to the wider M. tuberculosis complex (MTC) diversity, we analyzed the genomes of 1,974 previously published MTC strains (14). This identified a single T80A+A90G double mutant, which, as expected, resulted in a false-positive result with the MTBDR*sl* assay (Table 1, C00014838). We then analyzed this strain in a wider collection of 94 Uganda or Uganda-like strains, including 27 T80A+A90G double mutants (or variants thereof), which confirmed that this double mutation was a marker for a subgroup of Uganda strains (Fig. 2; see also Table S1 in the supplemental material). Of these 28 double mutant strains (or variants thereof), 25 originated from the Democratic Republic of Congo in a study of acquired drug resistance, nested in routine surveillance conducted from 2006 to 2009 for drug resistance in Kinshasa (18). Specifically, strains were drawn from a collection of 324 phenotypically rifampin-resistant isolates, resulting in a frequency of 7.7% (25/324), which is in line with the aforementioned frequency of 7.2% in Kinshasa during the period of 2011 to 2013 (8).

**TABLE 1:** MTBDR*sl gyrA* probe results for clinical strains and plasmids^*a*^

Strain/plasmid name	*gyrA* mutation(s)	WT1	WT2	WT3	MUT1	MUT2	MUT3A	MUT3B	MUT3C	MUT3D	Comment	Interpretation of result
C00014838	Acc/Gcc T80A, gCg/gGg A90G	X		X							WT2 binding prevented	False resistant
C00008711	caC/caT H85H	X	X	X								True susceptible
C00011395	gcG/gcA A90A^*b*^	X		X							WT2 binding prevented	False resistant
C00005422^*c*^ and C00005429^*c*^	atC/atT I92I	X									WT2 and WT3 binding prevented	False resistant
4312-12^*d*^	gaC/gaT D94D	X	X								WT3 binding prevented	False resistant
C00012906	ctG/ctA L96L	X	X	X								True susceptible
7 Colombian isolates^*e*^	Ctg/Ttg L96L	X	X								WT3 binding prevented	False resistant^*e*^
Plasmid 1	Wild type^*f*^	X	X	X							Negative control	True susceptible
Plasmid 2	aGc/aCc S95T^*g*^	X	X	X							Negative control	True susceptible
Plasmid 3	gCg/gTg A90V	X		X	X						WT2 and MUT1 control	True resistant
Plasmid 4	Tcg/Ccg S91P	X		X		X					WT2 and MUT2 control	True resistant
Plasmid 5	gAc/gCc D94A	X	X				X				WT3 and MUT3A control	True resistant
Plasmid 6	Gac/Aac D94N	X	X					X			WT3 and MUT3B control	True resistant
Plasmid 7	Gac/Tac D94Y	X	X								WT3 and MUT3B control, but MUT3B failed to bind	True resistant, but D94Y not identified^*h*^
Plasmid 8	gAc/gGc D94G	X	X						X		WT3 and MUT3C control	True resistant
Plasmid 9	Gac/Cac D94H	X	X							X	WT4 and MUT3D control	True resistant
Plasmid 10	Acc/Gcc T80A, gCg/gGg A90G	X		X							WT2 binding prevented; agreement with C00014838	False resistant
Plasmid 10a	Acc/Gcc T80A, gCg/gGg A90G, Tcg/Ccg S91P	X		X							WT2 and MUT2 binding prevented	True resistant,^*i*^ but S91P mutation not identified
Plasmid 11	gcG/gcA A90A	X		X							WT2 binding prevented, agreement with C00011395	False resistant
Plasmid 11a	gcG/gcA A90A, Tcg/Ccg S91P	X		X							WT2 and MUT2 binding prevented	True resistant, but S91P not identified
Plasmid 11b	gCG/gTA A90V^*j*^	X		X							WT2 binding prevented	True resistant, but A90V not identified
Plasmid 12	atC/atT I92I	X									WT2 and WT3 binding prevented; agreement with C00005422 and C00005429	False resistant
Plasmid 12a	Tcg/Ccg S91P, atC/atT I92I	X									WT2 and MUT2 binding prevented	True resistant, but S91P not identified

a Unless otherwise stated, testing was done with version 1 of the assay. WT or MUT bands (Fig. 1) were deemed positive if they were as strong as or stronger than the amplification control band, as stipulated in the instructions for use (24, 40). Plasmids were used to investigate combinations of mutations that could arise but, to our knowledge, have not been reported to date. In this context, plasmids 1 to 12 served as controls to demonstrate that plasmids could be used instead of genomic DNA. Plasmids 10a, 11a, 11b, and 12a indicate that the known A90V or S91P resistance mutations were detected but not identified by the corresponding mutant probes in the T80A+A90G, A90A, or I92I strain background. It should be noted, however, that if the strain population is not homogeneous, the effects of these mutations may differ from those simulated in these experiments (see Supplemental Methods in the supplemental material).

b Also observed in a strain from China (44).

c The two samples were from the same patient.

d Tested with version 2 of the assay.

e One strain had a D94G minority mutation, which resulted in the binding of probe MUT3C. In this case, this was not a false-resistant result.

f H37Rv reference sequence.

g Ser at codon 95 is an H37Rv-specific mutation (17). All subsequent *gyrA* plasmids have the aGc/aCc S95T change. The *gyrA* Gag/Cag E21Q polymorphism was not taken into consideration, since it lay outside the area targeted by probes, as shown in Fig. 1 (45).

h MUT3B did not identify D94Y, contrary to the package insert (24). This was in agreement with observations from other studies that used version 1 or 2 of the assay (1, 9, 23, 46–49), although the mutation was identified in some cases (1).

i Assuming that the S91P mutation causes resistance in a T80A+A90G background, which is not necessarily the case, as discussed in the Fig. 2 legend.

j A90V mutation in a gcG/gcA A90A background.

Synonymous mutations have been shown in other contexts to cause systematic false-positive results, such as those for rifampin when using genotypic DST assays such as the Hain GenoType MTBDR*plus* or Cepheid Xpert MTB/RIF (19, 20). To date, the equivalent phenomenon had not been described with the MTBDR*sl* assay. We therefore screened the aforementioned 1,974 genomes and the Sanger sequencing data of 104 MDR TB strains from Medellín (Colombia) and unpublished data, which identified six different synonymous mutations in the fluoroquinolone resistance-determining region of *gyrA* (14, 21). Two of the synonymous mutations (caC/caT H85H and ctG/ctA L96L) did not cause false-resistance results by preventing the corresponding wild-type bands from binding (Table 1). In contrast, the remaining four did, including a mutation at another nucleotide position of codon 96 (Ctg/Ttg) (Table 1), which was found in seven Haarlem strains from Colombia that were closely related based on 24-locus MIRU-VNTR, resulting in a systematic false-resistance rate of 6.7% (7/104) in Medellín.

Furthermore, we showed that the T80A+A90G double mutations and the synonymous gcG/gcA A90A and atC/atT I92I mutations prevented the binding of not only their corresponding wild-type band(s) but also that of the Tcg/Ccg S91P probe (Table 1). Similarly, if the A90V resistance mutation arose in the A90A background (i.e., by a further change in the triplet gCG/gTA), it would not be detected by the gCg/gTg A90V probe.

The consequences of these findings depend on a variety of factors. The aforementioned mutations that result in systematic false-positive results are likely rare globally (i.e., <1% based on the total number of strains initially screened for this study). Nevertheless, they can be frequent locally. Synonymous mutations in particular are not selected against, which means that it is only a matter of time until the MTBDR*sl* is used in a region where it has a poor positive predictive value, as would be the case in Medellín. As a result, the absence of binding of wild-type probes without concomitant binding of a mutant probe is a true marker of resistance in most settings, because this binding pattern identifies (i) valid resistance mutations, such as G88C and G88A, that can be inferred only by the absence of WT1, (ii) D94Y, which, contrary to the package insert, was not detected by MUT3B (Table 1), and (iii) mutations that are targeted by specific mutant probes but to which the mutant probes do not bind for unknown reasons (i.e., when the absence of wild-type probes acts as a failsafe method) (22, 23). In other words, simply ignoring wild-type bands would likely result in a significant loss of MTBDR*sl* sensitivity.

In the MTBDR*sl* instructions, Hain acknowledges that synonymous mutations can result in false-resistant results, but the instructions do not comment on the T80A+A90G mutation or on the effects of synonymous and nonsynonymous mutations on the binding of mutant probes (24). The WHO report that endorsed the assay did not discuss the consequences of systematic false-resistant results (3, 4). In light of the potentially severe consequences of systematic false-resistance results, we propose that in cases where fluoroquinolone resistance is inferred from the absence of a wild-type band alone, appropriate confirmatory testing is undertaken immediately. This would not only be beneficial to the patient but also may prove cost-effective overall for the TB control program (i.e., by avoiding the unnecessary use of more toxic, less effective, and often more expensive drugs, thereby minimizing transmission and enabling preventive therapy of contacts with fluoroquinolones [9, 25]). Given that systematic false-positives are rare in most settings, we would advise not discontinuing fluoroquinolone treatment while confirmatory testing is being carried out, provided this testing is done rapidly (e.g., using targeted sequencing of the locus in question to identify synonymous mutations, the T80A+A90G mutations, or any resistance mutations). Ideally, this should be complemented with phenotypic DST to identify heteroresistance that is missed by Sanger sequencing, which cannot detect mutations that occur in below 10 to 15% of the total population (26). Alternatively, fluoroquinolones could be kept in the regimen but not counted as an effective agent until systematic false-positives are excluded.

Although not investigated here, these highlighted issues likely apply to some, if not all, other commercial genotypic DST assays for fluoroquinolones, which are manufactured by Autoimmun Diagnostika, NIPRO, Seegene, YD Diagnostics, and Zeesan Biotech (27–32). Our findings therefore underline the need for diagnostic companies, including Cepheid, which is currently adapting its GeneXpert system for fluoroquinolone testing, to consider the genetic diversity within the MTC at the development stage and to monitor test performance after uptake in clinical settings (19, 33, 34). Importantly, this also applies to software tools designed to automate the analysis of whole-genome sequencing data. In fact, three of the current tools (KvarQ, Mykrobe Predictor TB, and TB Profiler) misclassified strain BTB-08-045 with *gyrA* T80A+A90G as resistant to at least one fluoroquinolone because the respective mutation catalogues of these tools list A90G as a resistance mutation, whereas the tools CASTB and PhyResSE correctly classified the strain (35–39).

## Supplementary Material

Supplemental material
