# Supplementary material for: Some Synonymous and Nonsynonymous gyrA Mutations in Mycobacterium tuberculosis Lead to Systematic False-Positive Fluoroquinolone Resistance Results with the Hain GenoType MTBDRsl Assays
Source: Antimicrob Agents Chemother. 2017 Mar 24;61(4):e02169-16. doi: 10.1128/AAC.02169-16 (PMC5365657; doi:10.1128/AAC.02169-16)
Supplement: Supplemental material [file supp_61_4_e02169-16__index.html]

Supplemental material 

# Some Synonymous and Nonsynonymous *gyrA* Mutations in Mycobacterium tuberculosis Lead to Systematic False-Positive Fluoroquinolone Resistance Results with the Hain GenoType MTBDR*sl* Assays

## Supplemental material

- Supplemental file 1 -

  Supplemental methods

  PDF, 96K
- Supplemental file 2 -

  Supplemental Table S1

  XLSX, 16K
