# Supplementary material for: Some Synonymous and Nonsynonymous gyrA Mutations in Mycobacterium tuberculosis Lead to Systematic False-Positive Fluoroquinolone Resistance Results with the Hain GenoType MTBDRsl Assays
Source: Antimicrob Agents Chemother. 2017 Mar 24;61(4):e02169-16. doi: 10.1128/AAC.02169-16 (PMC5365657; doi:10.1128/AAC.02169-16)
Supplement: Supplemental material [file AAC.02169-16_zac004176042s1.pdf]

Supplementary comments for Table 1

Because of the different limits of detection of WT vs. MUT probes, the effects of the mutations in plasmids 7, 10a, 11a-b, and 12a likely depend on whether a patient harbours a homogeneous strain population, as simulated in these experiments, or has a mixed infection. These limits have not been explored for the MTBDR*sl* assays, but assuming that they are similar to the ones for the Hain Genotype MTBDR*plus* assay, this would mean that a resistance mutation is identified by a MUT probe at about 5% of the total population and, conversely, that binding of a WT probe is only prevented if the mutation in question is present at 95-100% of the population (1, 2). Therefore, if a patient had, for example, a 50:50 infection of a fully wild-type strain with a strain of genotype 10a, a false-susceptible result would likely arise, although this remains to be shown experimentally.

Ethics approvals and source of strains

Colombia: The use of Colombian isolates in the study was approved by institutional ethical committee from Corporación para Investigaciones Biológicas since confidentiality of the original patient name was maintained and no risk was derived from the study.

Democratic Republic of Congo: The institutional review body of the Institute of Tropical Medicine approved the analysis of anonymised, stored mycobacteria.

Germany: The molecular epidemiological studies were embedded in mandatory routine surveillance and contact investigation work performed by the public health offices according to the legal mandate of the German Infection Protection Act. They were approved by the Hamburg and Schleswig-Holstein Commissioners for Data Protection (3).

Uganda: DNA samples were isolated as part of the routine diagnostic service from HIV-positive and HIV-negative TB patients in Mbarara, Uganda and were included in a previously published molecular epidemiological study (4).

UK: Approval for whole-genome sequencing and analysis was granted by the UK National Research Ethics Service (12/EE/0439) and the Cambridge University Hospitals NHS Foundation Trust R&D Department (A092685).

Sierra Leone: DNA samples from Sierra Leone were derived from a drug resistance survey in the Western Area and Kenema districts including all smear positive cases registered for re-treatment between March 2003 and June 2004. Patients were interviewed thoroughly and informed consent was obtained (5).

South Africa: Not required as these data have been published previously (3).

Sweden: Not required.

Materials and methods

Resistance survey from Democratic Republic of Congo

Sputum from patients with treatment failure or relapse was shipped in cetylpyridinium chloride to Antwerp for culture and drug-susceptibility testing to 1st and 2nd line drugs. Drug-susceptibility testing for rifampicin was carried out at 40 mg/L on Löwenstein-Jensen medium using the proportion method.

Fluoroquinolone drug-susceptibility testing

Belgium: Testing for ofloxacin was carried out at 2 mg/L on Middlebrook 7H11 medium using the proportion method.

Colombia: Testing was conducted as previously described (6).

South Africa and UK: Testing was carried out with the BACTEC MGIT 960 at 2 mg/L for ofloxacin and 0.5 mg/L and 2 mg/L for moxifloxacin.

Sweden: Antimicrobial susceptibility testing was performed in Middlebrook 7H10 media as previously described (7).

All results are shown in Table S1.

Hain GenoType MTBDRsl testing

Version 1 or 2 were used according to the manufacturer's instructions on cultured isolates, whereas only version 1 was used to analyse the plasmids with inserts of *gyrA* fragments in a pUC57 plasmid

backbone (GenScript, Piscataway, NJ, USA) (8, 9). Specifically, the freeze-dried plasmids (4 µg) were resuspended in 1 mL of sterile distilled water and a 1/1000 dilution of the stock was used as starting material for testing.

Whole-genome sequencing and phylogenetic analysis

DNA libraries were prepared with the Nextera XT kit and run on Illumina next generation sequencing platforms (MiSeq, Nextseq) as instructed by the manufacturer (Illumina, San Diego, USA). Reads were mapped to the genome of the *M. tuberculosis* reference strain H37Rv (GenBank ID: NC_000962.3) with the exact alignment program SARUMAN (10). Single nucleotide polymorphisms (SNPs) with a minimum coverage of 10x and 75% allele frequency were called by customized perl scripts, excluding positions with SNPs in repetitive regions and in genes associated with drug resistance. The remaining positions that met the above threshold levels in at least 95% of all isolates were considered as valid and combined for all isolates and used for a concatenated sequence alignment. Raw data (fastq files) was submitted to the European Nucleotide Archive (ENA) (Table S1).

A maximum likelihood tree was calculated, based on the concatenated sequence alignment, using FastTree with a general time reversible (GTR) substituition model, 1,000 resamples and Gamma20 likelihood optimization to account for rate heterogeneity among sites (11).

Comparison of automated software tools to analyses WGS data

The WGS data of BTB-08-045, which was used as a representative fluoroquinolone-susceptible strain with the *gyrA* T80A+A90G mutations (Table S1), were analyzed with CASTB, KvarQ (version 0.12.2 with the testsuites MTBC/phylo and MTBC/resistance), MykrobePredictor TB (0.1.3), PhyResSE (version 1.0 with version 27 of SNP catalogue), and TBProfiler (12-16).

GyrA plasmid sequences

Plasmid 1: wild-type

ACGGTCTGCTGGAGGCGGGGCTGAAGGCCGGGAAGAAGATCAACAAGGAAGACGGCATTCAGCGGTACAA
GGGTCTAGGTGAAATGGACGCTAAGGAGTTGTGGGAGACCACCATGGATCCCTCGGTTCGTGTGTTGCGT
CAAGTGACGCTGGACGACGCCGCCGCCGCCGACGAGTTGTTCTCCATCCTGATGGGCGAGGACGTCGACG
CGCGGCGCAGCTTTATCACCCGCAACGCCAAGGATGTTCGGTTCCTGGATGTCTAACGCAACCCTGCGTT
CGATTGCAAACGAGGAATAGATGACAGACACGACGTTGCCGCCTGACGACTCGCTCGACCGGATCGAACC
GGTTGACATCGAGCAGGAGATGCAGCGCAGCTACATCGACTATGCGATGAGCGTGATCGTCGGCCGCGCG
CTGCCGGAGGTGCGCGACGGGCTCAAGCCCGTGCATCGCCGGGTGCTCTATGCAATGTTCGATTCCGGCT
TCCGCCCGGACCGCAGCCACGCCAAGTCGGCCCGGTCGGTTGCCGAGACCATGGGCAACTACCACCCGCA
CGGCGACGCGTCGATCTACGACAGCCTGGTGCGCATGGCCCAGCCCTGGTCGCTGCGCTACCCGCTGGTG
GACGGCCAGGGCAACTTCGGCTCGCCAGGCAATGACCCACCGGCGGCGATGAGGTACACCGAAGCCCGGC
TGACCCCGTTGGCGATGGAGATGCTGAGGGAAATCGACGAGGAGACAGTCGATTTCATCCCTAACTACGA
CGGCCGGGTGCAAGAGCCGACGGTGCTACCCAGCCGGTTCCCCAACCTGCTGGCCAACGGGTCAGGCGGC
ATCGCGGTCGGCATGGCAACCAATATCCCGCCGCACAACCTGCGTGAGCTGGCCGACGCGGTGTTCTGGG
CGCTGGAGAATCACGACGCCGACGAAGAGGAGACCCTGGCCGCGGTCATGGGGCGGGTTAAAGGCCCGGA
CTTCCCGACCGCCGGACTGA

Plasmid 2: aGc/aCc S95T

ACGGTCTGCTGGAGGCGGGGCTGAAGGCCGGGAAGAAGATCAACAAGGAAGACGGCATTCAGCGGTACAA
GGGTCTAGGTGAAATGGACGCTAAGGAGTTGTGGGAGACCACCATGGATCCCTCGGTTCGTGTGTTGCGT
CAAGTGACGCTGGACGACGCCGCCGCCGCCGACGAGTTGTTCTCCATCCTGATGGGCGAGGACGTCGACG
CGCGGCGCAGCTTTATCACCCGCAACGCCAAGGATGTTCGGTTCCTGGATGTCTAACGCAACCCTGCGTT
CGATTGCAAACGAGGAATAGATGACAGACACGACGTTGCCGCCTGACGACTCGCTCGACCGGATCGAACC
GGTTGACATCGAGCAGGAGATGCAGCGCAGCTACATCGACTATGCGATGAGCGTGATCGTCGGCCGCGCG
CTGCCGGAGGTGCGCGACGGGCTCAAGCCCGTGCATCGCCGGGTGCTCTATGCAATGTTCGATTCCGGCT
TCCGCCCGGACCGCAGCCACGCCAAGTCGGCCCGGTCGGTTGCCGAGACCATGGGCAACTACCACCCGCA
CGGCGACGCGTCGATCTACGACA**C**CCTGGTGCGCATGGCCCAGCCCTGGTCGCTGCGCTACCCGCTGGTG
GACGGCCAGGGCAACTTCGGCTCGCCAGGCAATGACCCACCGGCGGCGATGAGGTACACCGAAGCCCGGC
TGACCCCGTTGGCGATGGAGATGCTGAGGGAAATCGACGAGGAGACAGTCGATTTCATCCCTAACTACGA
CGGCCGGGTGCAAGAGCCGACGGTGCTACCCAGCCGGTTCCCCAACCTGCTGGCCAACGGGTCAGGCGGC
ATCGCGGTCGGCATGGCAACCAATATCCCGCCGCACAACCTGCGTGAGCTGGCCGACGCGGTGTTCTGGG
CGCTGGAGAATCACGACGCCGACGAAGAGGAGACCCTGGCCGCGGTCATGGGGCGGGTTAAAGGCCCGGA
CTTCCCGACCGCCGGACTGA

Plasmid 3: gCg/gTg A90V, aGc/aCc S95T

ACGGTCTGCTGGAGGCGGGGCTGAAGGCCGGGAAGAAGATCAACAAGGAAGACGGCATTCAGCGGTACAA
GGGTCTAGGTGAAATGGACGCTAAGGAGTTGTGGGAGACCACCATGGATCCCTCGGTTCGTGTGTTGCGT
CAAGTGACGCTGGACGACGCCGCCGCCGCCGACGAGTTGTTCTCCATCCTGATGGGCGAGGACGTCGACG
CGCGGCGCAGCTTTATCACCCGCAACGCCAAGGATGTTCGGTTCCTGGATGTCTAACGCAACCCTGCGTT
CGATTGCAAACGAGGAATAGATGACAGACACGACGTTGCCGCCTGACGACTCGCTCGACCGGATCGAACC
GGTTGACATCGAGCAGGAGATGCAGCGCAGCTACATCGACTATGCGATGAGCGTGATCGTCGGCCGCGCG
CTGCCGGAGGTGCGCGACGGGCTCAAGCCCGTGCATCGCCGGGTGCTCTATGCAATGTTCGATTCCGGCT
TCCGCCCGGACCGCAGCCACGCCAAGTCGGCCCGGTCGGTTGCCGAGACCATGGGCAACTACCACCCGCA
CGGCGACG**T**GTCGATCTACGACA**C**CCTGGTGCGCATGGCCCAGCCCTGGTCGCTGCGCTACCCGCTGGTG
GACGGCCAGGGCAACTTCGGCTCGCCAGGCAATGACCCACCGGCGGCGATGAGGTACACCGAAGCCCGGC
TGACCCCGTTGGCGATGGAGATGCTGAGGGAAATCGACGAGGAGACAGTCGATTTCATCCCTAACTACGA
CGGCCGGGTGCAAGAGCCGACGGTGCTACCCAGCCGGTTCCCCAACCTGCTGGCCAACGGGTCAGGCGGC
ATCGCGGTCGGCATGGCAACCAATATCCCGCCGCACAACCTGCGTGAGCTGGCCGACGCGGTGTTCTGGG
CGCTGGAGAATCACGACGCCGACGAAGAGGAGACCCTGGCCGCGGTCATGGGGCGGGTTAAAGGCCCGGA
CTTCCCGACCGCCGGACTGA

Plasmid 4: Tcg/Ccg S91P, aGc/aCc S95T

ACGGTCTGCTGGAGGCGGGGCTGAAGGCCGGGAAGAAGATCAACAAGGAAGACGGCATTCAGCGGTACAA
GGGTCTAGGTGAAATGGACGCTAAGGAGTTGTGGGAGACCACCATGGATCCCTCGGTTCGTGTGTTGCGT
CAAGTGACGCTGGACGACGCCGCCGCCGCCGACGAGTTGTTCTCCATCCTGATGGGCGAGGACGTCGACG
CGCGGCGCAGCTTTATCACCCGCAACGCCAAGGATGTTCGGTTCCTGGATGTCTAACGCAACCCTGCGTT
CGATTGCAAACGAGGAATAGATGACAGACACGACGTTGCCGCCTGACGACTCGCTCGACCGGATCGAACC
GGTTGACATCGAGCAGGAGATGCAGCGCAGCTACATCGACTATGCGATGAGCGTGATCGTCGGCCGCGCG
CTGCCGGAGGTGCGCGACGGGCTCAAGCCCGTGCATCGCCGGGTGCTCTATGCAATGTTCGATTCCGGCT
TCCGCCCGGACCGCAGCCACGCCAAGTCGGCCCGGTCGGTTGCCGAGACCATGGGCAACTACCACCCGCA
CGGCGACGCG**C**CGATCTACGACA**C**CCTGGTGCGCATGGCCCAGCCCTGGTCGCTGCGCTACCCGCTGGTG
GACGGCCAGGGCAACTTCGGCTCGCCAGGCAATGACCCACCGGCGGCGATGAGGTACACCGAAGCCCGGC
TGACCCCGTTGGCGATGGAGATGCTGAGGGAAATCGACGAGGAGACAGTCGATTTCATCCCTAACTACGA
CGGCCGGGTGCAAGAGCCGACGGTGCTACCCAGCCGGTTCCCCAACCTGCTGGCCAACGGGTCAGGCGGC
ATCGCGGTCGGCATGGCAACCAATATCCCGCCGCACAACCTGCGTGAGCTGGCCGACGCGGTGTTCTGGG

CGCTGGAGAATCACGACGCCGACGAAGAGGAGACCCTGGCCGCGGTCATGGGGCGGGTTAAAGGCCCGGA
CTTCCCGACCGCCGGACTGA

Plasmid 5: gAc/gCc D94A, aGc/aCc S95T
ACGGTCTGCTGGAGGCGGGGCTGAAGGCCGGGAAGAAGATCAACAAGGAAGACGGCATTCAGCGGTACAA
GGGTCTAGGTGAAATGGACGCTAAGGAGTTGTGGGAGACCACCATGGATCCCTCGGTTCGTGTGTTGCGT
CAAGTGACGCTGGACGACGCCGCCGCCGCCGACGAGTTGTTCTCCATCCTGATGGGCGAGGACGTCGACG
CGCGGCGCAGCTTTATCACCCGCAACGCCAAGGATGTTCGGTTCCTGGATGTCTAACGCAACCCTGCGTT
CGATTGCAAACGAGGAATAGATGACAGACACGACGTTGCCGCCTGACGACTCGCTCGACCGGATCGAACC
GGTTGACATCGAGCAGGAGATGCAGCGCAGCTACATCGACTATGCGATGAGCGTGATCGTCGGCCCGCGCG
CTGCCGGAGGTGCGCGACGGGCTCAAGCCCGTGCATCGCCGGGTGCTCTATGCAATGTTCGATTCCGGCT
TCCGCCCGGACCGCAGCCACGCCAAGTCGGCCCGGTCGGTTGCCGAGACCATGGGCAACTACCACCCGCA
CGGCGACGCGTCGATCTACG**C**CA**C**CCTGGTGCGCATGGCCCAGCCCTGGTCGCTGCGCTACCCGCTGGTG
GACGGCCAGGGCAACTTCGGCTCGCCAGGCAATGACCCACCGGCGGCGATGAGGTACACCGAAGCCCGGC
TGACCCCGTTGGCGATGGAGATGCTGAGGGAAATCGACGAGGAGACAGTCGATTTCATCCCTAACTACGA
CGGCCGGGTGCAAGAGCCGACGGTGCTACCCAGCCGGTTCCCCAACCTGCTGGCCAACGGGTCAGGCGGC
ATCGCGGTCGGCATGGCAACCAATATCCCGCCGCACAACCTGCGTGAGCTGGCCGACGCGGTGTTCTGGG
CGCTGGAGAATCACGACGCCGACGAAGAGGAGACCCTGGCCGCGGTCATGGGGCGGGTTAAAGGCCCGGA
CTTCCCGACCGCCGGACTGA

Plasmid 6: Gac/Aac D94N, aGc/aCc S95T
ACGGTCTGCTGGAGGCGGGGCTGAAGGCCGGGAAGAAGATCAACAAGGAAGACGGCATTCAGCGGTACAA
GGGTCTAGGTGAAATGGACGCTAAGGAGTTGTGGGAGACCACCATGGATCCCTCGGTTCGTGTGTTGCGT
CAAGTGACGCTGGACGACGCCGCCGCCGCCGACGAGTTGTTCTCCATCCTGATGGGCGAGGACGTCGACG
CGCGGCGCAGCTTTATCACCCGCAACGCCAAGGATGTTCGGTTCCTGGATGTCTAACGCAACCCTGCGTT
CGATTGCAAACGAGGAATAGATGACAGACACGACGTTGCCGCCTGACGACTCGCTCGACCGGATCGAACC
GGTTGACATCGAGCAGGAGATGCAGCGCAGCTACATCGACTATGCGATGAGCGTGATCGTCGGCCCGCGCG
CTGCCGGAGGTGCGCGACGGGCTCAAGCCCGTGCATCGCCGGGTGCTCTATGCAATGTTCGATTCCGGCT
TCCGCCCGGACCGCAGCCACGCCAAGTCGGCCCGGTCGGTTGCCGAGACCATGGGCAACTACCACCCGCA
CGGCGACGCGTCGATCTAC**A**ACA**C**CCTGGTGCGCATGGCCCAGCCCTGGTCGCTGCGCTACCCGCTGGTG
GACGGCCAGGGCAACTTCGGCTCGCCAGGCAATGACCCACCGGCGGCGATGAGGTACACCGAAGCCCGGC
TGACCCCGTTGGCGATGGAGATGCTGAGGGAAATCGACGAGGAGACAGTCGATTTCATCCCTAACTACGA
CGGCCGGGTGCAAGAGCCGACGGTGCTACCCAGCCGGTTCCCCAACCTGCTGGCCAACGGGTCAGGCGGC
ATCGCGGTCGGCATGGCAACCAATATCCCGCCGCACAACCTGCGTGAGCTGGCCGACGCGGTGTTCTGGG
CGCTGGAGAATCACGACGCCGACGAAGAGGAGACCCTGGCCGCGGTCATGGGGCGGGTTAAAGGCCCGGA
CTTCCCGACCGCCGGACTGA

Plasmid 7: Gac/Tac D94Y, aGc/aCc S95T
ACGGTCTGCTGGAGGCGGGGCTGAAGGCCGGGAAGAAGATCAACAAGGAAGACGGCATTCAGCGGTACAA
GGGTCTAGGTGAAATGGACGCTAAGGAGTTGTGGGAGACCACCATGGATCCCTCGGTTCGTGTGTTGCGT
CAAGTGACGCTGGACGACGCCGCCGCCGCCGACGAGTTGTTCTCCATCCTGATGGGCGAGGACGTCGACG
CGCGGCGCAGCTTTATCACCCGCAACGCCAAGGATGTTCGGTTCCTGGATGTCTAACGCAACCCTGCGTT
CGATTGCAAACGAGGAATAGATGACAGACACGACGTTGCCGCCTGACGACTCGCTCGACCGGATCGAACC
GGTTGACATCGAGCAGGAGATGCAGCGCAGCTACATCGACTATGCGATGAGCGTGATCGTCGGCCCGCGCG
CTGCCGGAGGTGCGCGACGGGCTCAAGCCCGTGCATCGCCGGGTGCTCTATGCAATGTTCGATTCCGGCT
TCCGCCCGGACCGCAGCCACGCCAAGTCGGCCCGGTCGGTTGCCGAGACCATGGGCAACTACCACCCGCA
CGGCGACGCGTCGATCTAC**T**ACA**C**CCTGGTGCGCATGGCCCAGCCCTGGTCGCTGCGCTACCCGCTGGTG
GACGGCCAGGGCAACTTCGGCTCGCCAGGCAATGACCCACCGGCGGCGATGAGGTACACCGAAGCCCGGC
TGACCCCGTTGGCGATGGAGATGCTGAGGGAAATCGACGAGGAGACAGTCGATTTCATCCCTAACTACGA
CGGCCGGGTGCAAGAGCCGACGGTGCTACCCAGCCGGTTCCCCAACCTGCTGGCCAACGGGTCAGGCGGC
ATCGCGGTCGGCATGGCAACCAATATCCCGCCGCACAACCTGCGTGAGCTGGCCGACGCGGTGTTCTGGG
CGCTGGAGAATCACGACGCCGACGAAGAGGAGACCCTGGCCGCGGTCATGGGGCGGGTTAAAGGCCCGGA
CTTCCCGACCGCCGGACTGA

Plasmid 8: gAc/gGc D94G, aGc/aCc S95T
ACGGTCTGCTGGAGGCGGGGCTGAAGGCCGGGAAGAAGATCAACAAGGAAGACGGCATTCAGCGGTACAA
GGGTCTAGGTGAAATGGACGCTAAGGAGTTGTGGGAGACCACCATGGATCCCTCGGTTCGTGTGTTGCGT
CAAGTGACGCTGGACGACGCCGCCGCCGCCGACGAGTTGTTCTCCATCCTGATGGGCGAGGACGTCGACG
CGCGGCGCAGCTTTATCACCCGCAACGCCAAGGATGTTCGGTTCCTGGATGTCTAACGCAACCCTGCGTT
CGATTGCAAACGAGGAATAGATGACAGACACGACGTTGCCGCCTGACGACTCGCTCGACCGGATCGAACC
GGTTGACATCGAGCAGGAGATGCAGCGCAGCTACATCGACTATGCGATGAGCGTGATCGTCGGCCCGCGCG
CTGCCGGAGGTGCGCGACGGGCTCAAGCCCGTGCATCGCCGGGTGCTCTATGCAATGTTCGATTCCGGCT
TCCGCCCGGACCGCAGCCACGCCAAGTCGGCCCGGTCGGTTGCCGAGACCATGGGCAACTACCACCCGCA
CGGCGACGCGTCGATCTACG**G**CA**C**CCTGGTGCGCATGGCCCAGCCCTGGTCGCTGCGCTACCCGCTGGTG
GACGGCCAGGGCAACTTCGGCTCGCCAGGCAATGACCCACCGGCGGCGATGAGGTACACCGAAGCCCGGC
TGACCCCGTTGGCGATGGAGATGCTGAGGGAAATCGACGAGGAGACAGTCGATTTCATCCCTAACTACGA

CGGCCGGGTGCAAGAGCCGACGGTGCTACCCAGCCGGTTCCCCAACCTGCTGGCCAACGGGTCAGGCGGC
ATCGCGGTCGGCATGGCAACCAATATCCCGCCGCACAACCTGCGTGAGCTGGCCGACGCGGTGTTCTGGG
CGCTGGAGAATCACGACGCCGACGAAGAGGAGACCCTGGCCGCGGTCATGGGGCGGGTTAAAGGCCCGGA
CTTCCCGACCGCCGGACTGA

Plasmid 9: Gac/Cac D94H, aGc/aCc S95T
ACGGTCTGCTGGAGGCGGGGCTGAAGGCCGGGAAGAAGATCAACAAGGAAGACGGCATTCAGCGGTACAA
GGGTCTAGGTGAAATGGACGCTAAGGAGTTGTGGGAGACCACCATGGATCCCTCGGTTCGTGTGTTGCGT
CAAGTGACGCTGGACGACGCCGCCGCCGCCGACGAGTTGTTCTCCATCCTGATGGGCGAGGACGTCGACG
CGCGGCGCAGCTTTATCACCCGCAACGCCAAGGATGTTCGGTTCCTGGATGTCTAACGCAACCCTGCGTT
CGATTGCAAACGAGGAATAGATGACAGACACGACGTTGCCGCCTGACGACTCGCTCGACCGGATCGAACC
GGTTGACATCGAGCAGGAGATGCAGCGCAGCTACATCGACTATGCGATGAGCGTGATCGTCGGCCGCGCG
CTGCCGGAGGTGCGCGACGGGCTCAAGCCCGTGCATCGCCGGGTGCTCTATGCAATGTTCGATTCCGGCT
TCCGCCCCGGACCGCAGCCACGCCAAGTCGGCCCGGTCGGTTGCCGAGACCATGGGCAACTACCACCCGCA
CGGCGACGCGTCGATCTAC**C**ACA**C**CCTGGTGCGCATGGCCCAGCCCTGGTCGCTGCGCTACCCGCTGGTG
GACGGCCAGGGCAACTTCGGCTCGCCAGGCAATGACCCACCGGCGGCGATGAGGTACACCGAAGCCCGGC
TGACCCCGTTGGCGATGGAGATGCTGAGGGAAATCGACGAGGAGACAGTCGATTTCATCCCTAACTACGA
CGGCCGGGTGCAAGAGCCGACGGTGCTACCCAGCCGGTTCCCCAACCTGCTGGCCAACGGGTCAGGCGGC
ATCGCGGTCGGCATGGCAACCAATATCCCGCCGCACAACCTGCGTGAGCTGGCCGACGCGGTGTTCTGGG
CGCTGGAGAATCACGACGCCGACGAAGAGGAGACCCTGGCCGCGGTCATGGGGCGGGTTAAAGGCCCGGA
CTTCCCGACCGCCGGACTGA

Plasmid 10: Acc/Gcc T80A, gCg/gGg A90G, aGc/aCc S95T
ACGGTCTGCTGGAGGCGGGGCTGAAGGCCGGGAAGAAGATCAACAAGGAAGACGGCATTCAGCGGTACAA
GGGTCTAGGTGAAATGGACGCTAAGGAGTTGTGGGAGACCACCATGGATCCCTCGGTTCGTGTGTTGCGT
CAAGTGACGCTGGACGACGCCGCCGCCGCCGACGAGTTGTTCTCCATCCTGATGGGCGAGGACGTCGACG
CGCGGCGCAGCTTTATCACCCGCAACGCCAAGGATGTTCGGTTCCTGGATGTCTAACGCAACCCTGCGTT
CGATTGCAAACGAGGAATAGATGACAGACACGACGTTGCCGCCTGACGACTCGCTCGACCGGATCGAACC
GGTTGACATCGAGCAGGAGATGCAGCGCAGCTACATCGACTATGCGATGAGCGTGATCGTCGGCCGCGCG
CTGCCGGAGGTGCGCGACGGGCTCAAGCCCGTGCATCGCCGGGTGCTCTATGCAATGTTCGATTCCGGCT
TCCGCCCCGGACCGCAGCCACGCCAAGTCGGCCCGGTCGGTTGCCGAG**G**CCATGGGCAACTACCACCCGCA
CGGCGACG**G**GTCGATCTACGACA**C**CCTGGTGCGCATGGCCCAGCCCTGGTCGCTGCGCTACCCGCTGGTG
GACGGCCAGGGCAACTTCGGCTCGCCAGGCAATGACCCACCGGCGGCGATGAGGTACACCGAAGCCCGGC
TGACCCCGTTGGCGATGGAGATGCTGAGGGAAATCGACGAGGAGACAGTCGATTTCATCCCTAACTACGA
CGGCCGGGTGCAAGAGCCGACGGTGCTACCCAGCCGGTTCCCCAACCTGCTGGCCAACGGGTCAGGCGGC
ATCGCGGTCGGCATGGCAACCAATATCCCGCCGCACAACCTGCGTGAGCTGGCCGACGCGGTGTTCTGGG
CGCTGGAGAATCACGACGCCGACGAAGAGGAGACCCTGGCCGCGGTCATGGGGCGGGTTAAAGGCCCGGA
CTTCCCGACCGCCGGACTGA

Plasmid 10a: Acc/Gcc T80A, gCg/gGg A90G, Tcg/Ccg S91P, aGc/aCc S95T
ACGGTCTGCTGGAGGCGGGGCTGAAGGCCGGGAAGAAGATCAACAAGGAAGACGGCATTCAGCGGTACAA
GGGTCTAGGTGAAATGGACGCTAAGGAGTTGTGGGAGACCACCATGGATCCCTCGGTTCGTGTGTTGCGT
CAAGTGACGCTGGACGACGCCGCCGCCGCCGACGAGTTGTTCTCCATCCTGATGGGCGAGGACGTCGACG
CGCGGCGCAGCTTTATCACCCGCAACGCCAAGGATGTTCGGTTCCTGGATGTCTAACGCAACCCTGCGTT
CGATTGCAAACGAGGAATAGATGACAGACACGACGTTGCCGCCTGACGACTCGCTCGACCGGATCGAACC
GGTTGACATCGAGCAGGAGATGCAGCGCAGCTACATCGACTATGCGATGAGCGTGATCGTCGGCCGCGCG
CTGCCGGAGGTGCGCGACGGGCTCAAGCCCGTGCATCGCCGGGTGCTCTATGCAATGTTCGATTCCGGCT
TCCGCCCCGGACCGCAGCCACGCCAAGTCGGCCCGGTCGGTTGCCGAG**G**CCATGGGCAACTACCACCCGCA
CGGCGACG**G**G**C**CGATCTACGACA**C**CCTGGTGCGCATGGCCCAGCCCTGGTCGCTGCGCTACCCGCTGGTG
GACGGCCAGGGCAACTTCGGCTCGCCAGGCAATGACCCACCGGCGGCGATGAGGTACACCGAAGCCCGGC
TGACCCCGTTGGCGATGGAGATGCTGAGGGAAATCGACGAGGAGACAGTCGATTTCATCCCTAACTACGA
CGGCCGGGTGCAAGAGCCGACGGTGCTACCCAGCCGGTTCCCCAACCTGCTGGCCAACGGGTCAGGCGGC
ATCGCGGTCGGCATGGCAACCAATATCCCGCCGCACAACCTGCGTGAGCTGGCCGACGCGGTGTTCTGGG
CGCTGGAGAATCACGACGCCGACGAAGAGGAGACCCTGGCCGCGGTCATGGGGCGGGTTAAAGGCCCGGA
CTTCCCGACCGCCGGACTGA

Plasmid 11: gcG/gcA A90A, aGc/aCc S95T
ACGGTCTGCTGGAGGCGGGGCTGAAGGCCGGGAAGAAGATCAACAAGGAAGACGGCATTCAGCGGTACAA
GGGTCTAGGTGAAATGGACGCTAAGGAGTTGTGGGAGACCACCATGGATCCCTCGGTTCGTGTGTTGCGT
CAAGTGACGCTGGACGACGCCGCCGCCGCCGACGAGTTGTTCTCCATCCTGATGGGCGAGGACGTCGACG
CGCGGCGCAGCTTTATCACCCGCAACGCCAAGGATGTTCGGTTCCTGGATGTCTAACGCAACCCTGCGTT
CGATTGCAAACGAGGAATAGATGACAGACACGACGTTGCCGCCTGACGACTCGCTCGACCGGATCGAACC
GGTTGACATCGAGCAGGAGATGCAGCGCAGCTACATCGACTATGCGATGAGCGTGATCGTCGGCCGCGCG
CTGCCGGAGGTGCGCGACGGGCTCAAGCCCGTGCATCGCCGGGTGCTCTATGCAATGTTCGATTCCGGCT
TCCGCCCCGGACCGCAGCCACGCCAAGTCGGCCCGGTCGGTTGCCGAGACCATGGGCAACTACCACCCGCA
CGGCGACGC**A**TCGATCTACGACA**C**CCTGGTGCGCATGGCCCAGCCCTGGTCGCTGCGCTACCCGCTGGTG

GACGGCCAGGGCAACTTCGGCTCGCCAGGCAATGACCCACCGGCGGCGATGAGGTACACCGAAGCCCGGC
TGACCCCGTTGGCGATGGAGATGCTGAGGGAAATCGACGAGGAGACAGTCGATTTCATCCCTAACTACGA
CGGCCGGGTGCAAGAGCCGACGGTGCTACCCAGCCGGTTCCCCAACCTGCTGGCCAACGGGTCAGGCGGC
ATCGCGGTCGGCATGGCAACCAATATCCCGCCGCACAACCTGCGTGAGCTGGCCGACGCGGTGTTCTGGG
CGCTGGAGAATCACGACGCCGACGAAGAGGAGACCCTGGCCGCGGTCATGGGGCGGGTTAAAGGCCCGGA
CTTCCCGACCGCCGGACTGA

Plasmid 11a: gcG/gcA A90A, Tcg/Ccg S91P, aGc/aCc S95T
ACGGTCTGCTGGAGGCGGGGCTGAAGGCCGGGAAGAAGATCAACAAGGAAGACGGCATTCAGCGGTACAA
GGGTCTAGGTGAAATGGACGCTAAGGAGTTGTGGGAGACCACCATGGATCCCTCGGTTCGTGTGTTGCGT
CAAGTGACGCTGGACGACGCCGCCGCCGCCGACGAGTTGTTCTCCATCCTGATGGGCGAGGACGTCGACG
CGCGGCGCAGCTTTATCACCCGCAACGCCAAGGATGTTCGGTTCCTGGATGTCTAACGCAACCCTGCGTT
CGATTGCAAACGAGGAATAGATGACAGACACGACGTTGCCGCCTGACGACTCGCTCGACCGGATCGAACC
GGTTGACATCGAGCAGGAGATGCAGCGCAGCTACATCGACTATGCGATGAGCGTGATCGTCGGCCGCGCG
CTGCCGGAGGTGCGCGACGGGCTCAAGCCCGTGCATCGCCGGGTGCTCTATGCAATGTTCGATTCCGGCT
TCCGCCCGGACCGCAGCCACGCCAAGTCGGCCCGGTCGGTTGCCGAGACCATGGGCAACTACCACCCGCA
CGGCGACGC**AC**CGATCTACGACA**C**CCTGGTGCGCATGGCCCAGCCCTGGTCGCTGCGCTACCCGCTGGTG
GACGGCCAGGGCAACTTCGGCTCGCCAGGCAATGACCCACCGGCGGCGATGAGGTACACCGAAGCCCGGC
TGACCCCGTTGGCGATGGAGATGCTGAGGGAAATCGACGAGGAGACAGTCGATTTCATCCCTAACTACGA
CGGCCGGGTGCAAGAGCCGACGGTGCTACCCAGCCGGTTCCCCAACCTGCTGGCCAACGGGTCAGGCGGC
ATCGCGGTCGGCATGGCAACCAATATCCCGCCGCACAACCTGCGTGAGCTGGCCGACGCGGTGTTCTGGG
CGCTGGAGAATCACGACGCCGACGAAGAGGAGACCCTGGCCGCGGTCATGGGGCGGGTTAAAGGCCCGGA
CTTCCCGACCGCCGGACTGA

Plasmid 11b: gCG/gTA A90V, aGc/aCc S95T
ACGGTCTGCTGGAGGCGGGGCTGAAGGCCGGGAAGAAGATCAACAAGGAAGACGGCATTCAGCGGTACAA
GGGTCTAGGTGAAATGGACGCTAAGGAGTTGTGGGAGACCACCATGGATCCCTCGGTTCGTGTGTTGCGT
CAAGTGACGCTGGACGACGCCGCCGCCGCCGACGAGTTGTTCTCCATCCTGATGGGCGAGGACGTCGACG
CGCGGCGCAGCTTTATCACCCGCAACGCCAAGGATGTTCGGTTCCTGGATGTCTAACGCAACCCTGCGTT
CGATTGCAAACGAGGAATAGATGACAGACACGACGTTGCCGCCTGACGACTCGCTCGACCGGATCGAACC
GGTTGACATCGAGCAGGAGATGCAGCGCAGCTACATCGACTATGCGATGAGCGTGATCGTCGGCCGCGCG
CTGCCGGAGGTGCGCGACGGGCTCAAGCCCGTGCATCGCCGGGTGCTCTATGCAATGTTCGATTCCGGCT
TCCGCCCGGACCGCAGCCACGCCAAGTCGGCCCGGTCGGTTGCCGAGACCATGGGCAACTACCACCCGCA
CGGCGACG**TA**TCGATCTACGACA**C**CCTGGTGCGCATGGCCCAGCCCTGGTCGCTGCGCTACCCGCTGGTG
GACGGCCAGGGCAACTTCGGCTCGCCAGGCAATGACCCACCGGCGGCGATGAGGTACACCGAAGCCCGGC
TGACCCCGTTGGCGATGGAGATGCTGAGGGAAATCGACGAGGAGACAGTCGATTTCATCCCTAACTACGA
CGGCCGGGTGCAAGAGCCGACGGTGCTACCCAGCCGGTTCCCCAACCTGCTGGCCAACGGGTCAGGCGGC
ATCGCGGTCGGCATGGCAACCAATATCCCGCCGCACAACCTGCGTGAGCTGGCCGACGCGGTGTTCTGGG
CGCTGGAGAATCACGACGCCGACGAAGAGGAGACCCTGGCCGCGGTCATGGGGCGGGTTAAAGGCCCGGA
CTTCCCGACCGCCGGACTGA

Plasmid 12: atC/atT I92I, aGc/aCc S95T
ACGGTCTGCTGGAGGCGGGGCTGAAGGCCGGGAAGAAGATCAACAAGGAAGACGGCATTCAGCGGTACAA
GGGTCTAGGTGAAATGGACGCTAAGGAGTTGTGGGAGACCACCATGGATCCCTCGGTTCGTGTGTTGCGT
CAAGTGACGCTGGACGACGCCGCCGCCGCCGACGAGTTGTTCTCCATCCTGATGGGCGAGGACGTCGACG
CGCGGCGCAGCTTTATCACCCGCAACGCCAAGGATGTTCGGTTCCTGGATGTCTAACGCAACCCTGCGTT
CGATTGCAAACGAGGAATAGATGACAGACACGACGTTGCCGCCTGACGACTCGCTCGACCGGATCGAACC
GGTTGACATCGAGCAGGAGATGCAGCGCAGCTACATCGACTATGCGATGAGCGTGATCGTCGGCCGCGCG
CTGCCGGAGGTGCGCGACGGGCTCAAGCCCGTGCATCGCCGGGTGCTCTATGCAATGTTCGATTCCGGCT
TCCGCCCGGACCGCAGCCACGCCAAGTCGGCCCGGTCGGTTGCCGAGACCATGGGCAACTACCACCCGCA
CGGCGACGCGTCGAT**T**TACGACA**C**CCTGGTGCGCATGGCCCAGCCCTGGTCGCTGCGCTACCCGCTGGTG
GACGGCCAGGGCAACTTCGGCTCGCCAGGCAATGACCCACCGGCGGCGATGAGGTACACCGAAGCCCGGC
TGACCCCGTTGGCGATGGAGATGCTGAGGGAAATCGACGAGGAGACAGTCGATTTCATCCCTAACTACGA
CGGCCGGGTGCAAGAGCCGACGGTGCTACCCAGCCGGTTCCCCAACCTGCTGGCCAACGGGTCAGGCGGC
ATCGCGGTCGGCATGGCAACCAATATCCCGCCGCACAACCTGCGTGAGCTGGCCGACGCGGTGTTCTGGG
CGCTGGAGAATCACGACGCCGACGAAGAGGAGACCCTGGCCGCGGTCATGGGGCGGGTTAAAGGCCCGGA
CTTCCCGACCGCCGGACTGA

Plasmid 12a: Tcg/Ccg S91P, atC/atT I92I, aGc/aCc S95T
ACGGTCTGCTGGAGGCGGGGCTGAAGGCCGGGAAGAAGATCAACAAGGAAGACGGCATTCAGCGGTACAA
GGGTCTAGGTGAAATGGACGCTAAGGAGTTGTGGGAGACCACCATGGATCCCTCGGTTCGTGTGTTGCGT
CAAGTGACGCTGGACGACGCCGCCGCCGCCGACGAGTTGTTCTCCATCCTGATGGGCGAGGACGTCGACG
CGCGGCGCAGCTTTATCACCCGCAACGCCAAGGATGTTCGGTTCCTGGATGTCTAACGCAACCCTGCGTT
CGATTGCAAACGAGGAATAGATGACAGACACGACGTTGCCGCCTGACGACTCGCTCGACCGGATCGAACC
GGTTGACATCGAGCAGGAGATGCAGCGCAGCTACATCGACTATGCGATGAGCGTGATCGTCGGCCGCGCG
CTGCCGGAGGTGCGCGACGGGCTCAAGCCCGTGCATCGCCGGGTGCTCTATGCAATGTTCGATTCCGGCT

TCCGCCCGGACCGCAGCCACGCCAAGTCGGCCCGGTCGGTTGCCGAGACCATGGGCAACTACCACCCGCA
CGGCGACGCG**C**CGAT**T**TACGACA**C**CCTGGTGCGCATGGCCCAGCCCTGGTCGCTGCGCTACCCGCTGGTG
GACGGCCAGGGCAACTTCGGCTCGCCAGGCAATGACCCACCGGCGGCGATGAGGTACACCGAAGCCCGGC
TGACCCCGTTGGCGATGGAGATGCTGAGGGAAATCGACGAGGAGACAGTCGATTTCATCCCTAACTACGA
CGGCCGGGTGCAAGAGCCGACGGTGCTACCCAGCCGGTTCCCCAACCTGCTGGCCAACGGGTCAGGCGGC
ATCGCGGTCGGCATGGCAACCAATATCCCGCCGCACAACCTGCGTGAGCTGGCCGACGCGGTGTTCTGGG
CGCTGGAGAATCACGACGCCGACGAAGAGGAGACCCTGGCCGCGGTCATGGGGCGGGTTAAAGGCCCGGA
CTTCCCGACCGCCGGACTGA

References

1. **Folkvardsen DB, Svensson E, Thomsen VO, Rasmussen EM, Bang D, Werngren J, Hoffner S, Hillemann D, Rigouts L.** 2013. Can molecular methods detect 1% isoniazid resistance in *Mycobacterium tuberculosis*? J Clin Microbiol **51:**1596-1599.
2. **Folkvardsen DB, Thomsen VO, Rigouts L, Rasmussen EM, Bang D, Bernaerts G, Werngren J, Toro JC, Hoffner S, Hillemann D, Svensson E.** 2013. Rifampin heteroresistance in *Mycobacterium tuberculosis* cultures as detected by phenotypic and genotypic drug susceptibility test methods. J Clin Microbiol **51:**4220-4222.
3. **Walker TM, Kohl TA, Omar SV, Hedge J, Del Ojo Elias C, Bradley P, Iqbal Z, Feuerriegel S, Niehaus KE, Wilson DJ, Clifton DA, Kapatai G, Ip CL, Bowden R, Drobniewski FA, Allix-Beguec C, Gaudin C, Parkhill J, Diel R, Supply P, Crook DW, Smith EG, Walker AS, Ismail N, Niemann S, Peto TE, Modernizing Medical Microbiology Informatics G.** 2015. Whole-genome sequencing for prediction of *Mycobacterium tuberculosis* drug susceptibility and resistance: a retrospective cohort study. Lancet Infect Dis **15:**1193-1202.
4. **Niemann S, Rüsch-Gerdes S, Joloba ML, Whalen CC, Guwatudde D, Ellner JJ, Eisenach K, Fumokong N, Johnson JL, Aisu T, Mugerwa RD, Okwera A, Schwander SK.** 2002. *Mycobacterium africanum* subtype II is associated with two distinct genotypes and is a major cause of human tuberculosis in Kampala, Uganda. J Clin Microbiol **40:**3398-3405.
5. **Homolka S, Post E, Oberhauser B, George AG, Westman L, Dafae F, Rüsch-Gerdes S, Niemann S.** 2008. High genetic diversity among *Mycobacterium tuberculosis* complex strains from Sierra Leone. BMC Microbiol **8:**103.
6. **Alvarez N, Zapata E, Mejia GI, Realpe T, Araque P, Pelaez C, Rouzaud F, Robledo J.** 2014. The structural modeling of the interaction between levofloxacin and the *Mycobacterium tuberculosis* gyrase catalytic site sheds light on the mechanisms of fluoroquinolones resistant tuberculosis in Colombian clinical isolates. Biomed Res Int **2014:**367268.
7. **Niward K, Ängeby K, Chryssanthou E, Paues J, Bruchfeld J, Juréen P, Giske CG, Kahlmeter G, Schön T.** 2016. Susceptibility testing breakpoints for *Mycobacterium tuberculosis* categorize isolates with resistance mutations in *gyrA* as susceptible to fluoroquinolones: implications for MDR-TB treatment and the definition of XDR-TB. J Antimicrob Chemother **71:**333-338.
8. **Hain Lifescience.** GenoType MTBDR*sl* VER 1.0. Instructions for use. 06/2015. IFU-317-06.
9. **Hain Lifescience.** GenoType MTBDR*sl* VER 2.0. Instructions for use 06/2015. IFU-317A-02.
10. **Blom J, Jakobi T, Doppmeier D, Jaenicke S, Kalinowski J, Stoye J, Goesmann A.** 2011. Exact and complete short-read alignment to microbial genomes using Graphics Processing Unit programming. Bioinformatics **27:**1351-1358.
11. **Price MN, Dehal PS, Arkin AP.** 2010. FastTree 2-approximately maximum-likelihood trees for large alignments. PLoS One **5:**e9490.
12. **Steiner A, Stucki D, Coscolla M, Borrell S, Gagneux S.** 2014. KvarQ: targeted and direct variant calling from fastq reads of bacterial genomes. BMC Genomics **15:**881.
13. **Bradley P, Gordon NC, Walker TM, Dunn L, Heys S, Huang B, Earle S, Pankhurst LJ, Anson L, de Cesare M, Piazza P, Votintseva AA, Golubchik T, Wilson DJ, Wyllie DH, Diel R, Niemann S, Feuerriegel S, Kohl TA, Ismail N, Omar SV, Smith EG, Buck D, McVean G, Walker AS, Peto TE, Crook DW, Iqbal Z.** 2015. Rapid antibiotic-resistance predictions from genome sequence data for *Staphylococcus aureus* and *Mycobacterium tuberculosis*. Nat Commun **6:**10063.
14. **Coll F, McNerney R, Preston MD, Guerra-Assuncao JA, Warry A, Hill-Cawthorne G, Mallard K, Nair M, Miranda A, Alves A, Perdigão J, Viveiros M, Portugal I, Hasan Z, Hasan R, Glynn JR, Martin N, Pain A, Clark TG.** 2015. Rapid determination of anti-tuberculosis drug resistance from whole-genome sequences. Genome Med **7:**51.
15. **Feuerriegel S, Schleusener V, Beckert P, Kohl TA, Miotto P, Cirillo DM, Cabibbe AM, Niemann S, Fellenberg K.** 2015. PhyResSE: web tool delineating *Mycobacterium tuberculosis* antibiotic resistance and lineage from whole-genome sequencing data. J Clin Microbiol **53:**1908-1914.
16. **Iwai H, Kato-Miyazawa M, Kirikae T, Miyoshi-Akiyama T.** 2015. CASTB (the Comprehensive Analysis Server for the *Mycobacterium tuberculosis* complex): A publicly accessible web server for epidemiological analyses, drug-resistance prediction and phylogenetic comparison of clinical isolates. Tuberculosis (Edinb) **95:**843-844.
